# “Stranger things” in the gut: uncommon items in gastrointestinal specimens

**DOI:** 10.1007/s00428-021-03188-1

**Published:** 2021-10-01

**Authors:** Federica Grillo, Michela Campora, Luca Carlin, Laura Cornara, Paola Parente, Alessandro Vanoli, Andrea Remo, Paola Migliora, Fiocca Roberto, Matteo Fassan, Luca Mastracci

**Affiliations:** 1 IRCCS Ospedale Policlinico San Martino, Anatomic Pathology Unit, Genova, Italy; 2 Anatomic Pathology, Department of Surgical Sciences and Integrated Diagnostics (DISC), University of Genova, Largo Rosanna Benzi 10, 16132 Genova, Italy; 3 Anatomic Pathology Section, S. Chiara Hospital, Trento, Italy; 4 Department for the Earth, Environment and Life Sciences (DiSTAV), University of Genoa, Genoa, Italy; 5 Pathology Unit, Fondazione IRCCS Ospedale Casa Sollievo Della Sofferenza, San Giovanni Rotondo, FG Italy; 6 Anatomic Pathology Unit, Department of Molecular Medicine, University of Pavia, and IRCCS San Matteo Hospital, Pavia, Italy; 7 Pathology Unit, Service Department, ULSS9 “Scaligera”, Verona, Italy; 8 Clinical Cytopathology Service and Pathology Institute of Locarno, Locarno, Switzerland; 9 Surgical Pathology Unit, Department of Medicine (DIMED), University of Padua, Padua, Italy; 10 Veneto Institute of Oncology IOV – IRCCS, Padua, Italy

**Keywords:** Parasites, Gastrointestinal diseases, Schistosomiasis, Strongyloides, Amebiasis

## Abstract

Organic (such as parasites or vegetable remnants) and inorganic substances may be encountered during routine pathology diagnostic work up of endoscopic gastrointestinal biopsy samples and major resections, causing possible diagnostic conundrums for the young and not so young pathologists. The main aim of this review is the description of the most frequent oddities one can encounter as foreign bodies, in gastrointestinal pathology, on the basis of the current literature and personal experience. The types of encountered substances are divided into four principal categories: parasites (helminths such as *Enterobius vermicularis*, *Strongyloides*, *Schistosoma*, and *Anisakis*, and protozoa such as *Entamoeba*, *Giardia* and some intestinal coccidia); drugs and pharmaceutical fillers (found as deposits and as bystanders, innocent or not); seeds (possibly confused with worms) and plant remnants; pollutants (secondary to post-resection or post-biopsy contamination of the sample). An ample library of images is provided in order to consent easy referencing for diagnostic routine.

## Introduction

Tissue samples taken from the luminal gut for the diagnosis of neoplastic or inflammatory conditions are some of the most frequent cases faced by an often overworked gastrointestinal-pathologist. Hopefully, only a few of these cases will pose diagnostic conundrums; foreign objects or foreign beings may cause such problems.

Various types of organic (such as parasites or vegetable remnants) and inorganic substances can be encountered in the gastrointestinal tract, and these may be difficult to identify for the pathologist. When this happens, the first step is to understand if the substance is contributory to the diagnosis, thus permitting the pathologist to recognize the causative agent of the disease, or if its presence is fortuitous and has no bearing on the disease at all; this distinction (causal and non-causal agents) can be intriguing and challenging (and not always possible).

The main aim of this review is the description of the most frequent oddities one can encounter in gastrointestinal pathology, on the basis of the current literature and personal experience and to provide ample reference images useful for routine diagnosis. Bacteria and viral infections have not been included in this review purposefully. With regard to bacterial infections, with notable exceptions such as *Helicobacter pylori*, morphology on hematoxylin and eosin (H&E)-stained slides of samples does not usually permit exact distinction of the pathogen and diagnosis is possible on stool culture. Some viral infections (e.g., Cytomegalovirus) lead to cytopathic effects which can be identified on H&E stained slides and usually require immunohistochemical confirmation.

From a practical point of view, 4 types of foreign matter can be found: (1) parasites; (2) drugs; (3) seeds/plant remnants; (4) substances that may pollute the sample during its processing.

## Parasites

### Helminths

The WHO reports that more than 1.5 billion people (24% of the world’s population) are infected by helminth infections worldwide, especially in tropical and subtropical areas. Notwithstanding this, gastro-intestinal pathologists encounter worms only rarely, at least in Western countries. Some species are found more frequently than others, and only these are discussed below.

### Enterobius vermicularis

Helminth ova or worms can be diagnosed not only by stool analysis, but they can be encountered in routine histology from surgical specimens or, rarely, endoscopic biopsies [1]. *E. vermicularis* are one of the most common human parasites, affecting about 200 million people worldwide, with children aged 5 ~ 10 years old accounting for over 30% of cases [2]. Traditionally named pinworms due to their vermiform, ivory white appearance, they live and reproduce from the ileum to the proximal colon. No symptoms are apparent until the females migrate to the anus, deposit their eggs and die, provoking a typical anal itch [3]. Occasionally, they can involve extraintestinal sites including the female genital tract [4].

In particular, larvae and adult pinworms can be seen in the appendix and they are associated with acute appendicitis between 0.2 and 41.8% of cases, with an overall average global prevalence of 4% in resected specimens [5]. They can be identified macroscopically (adult worms measure 1 cm length on average), but more often, they are seen under the microscope.

**Fig. 1:**
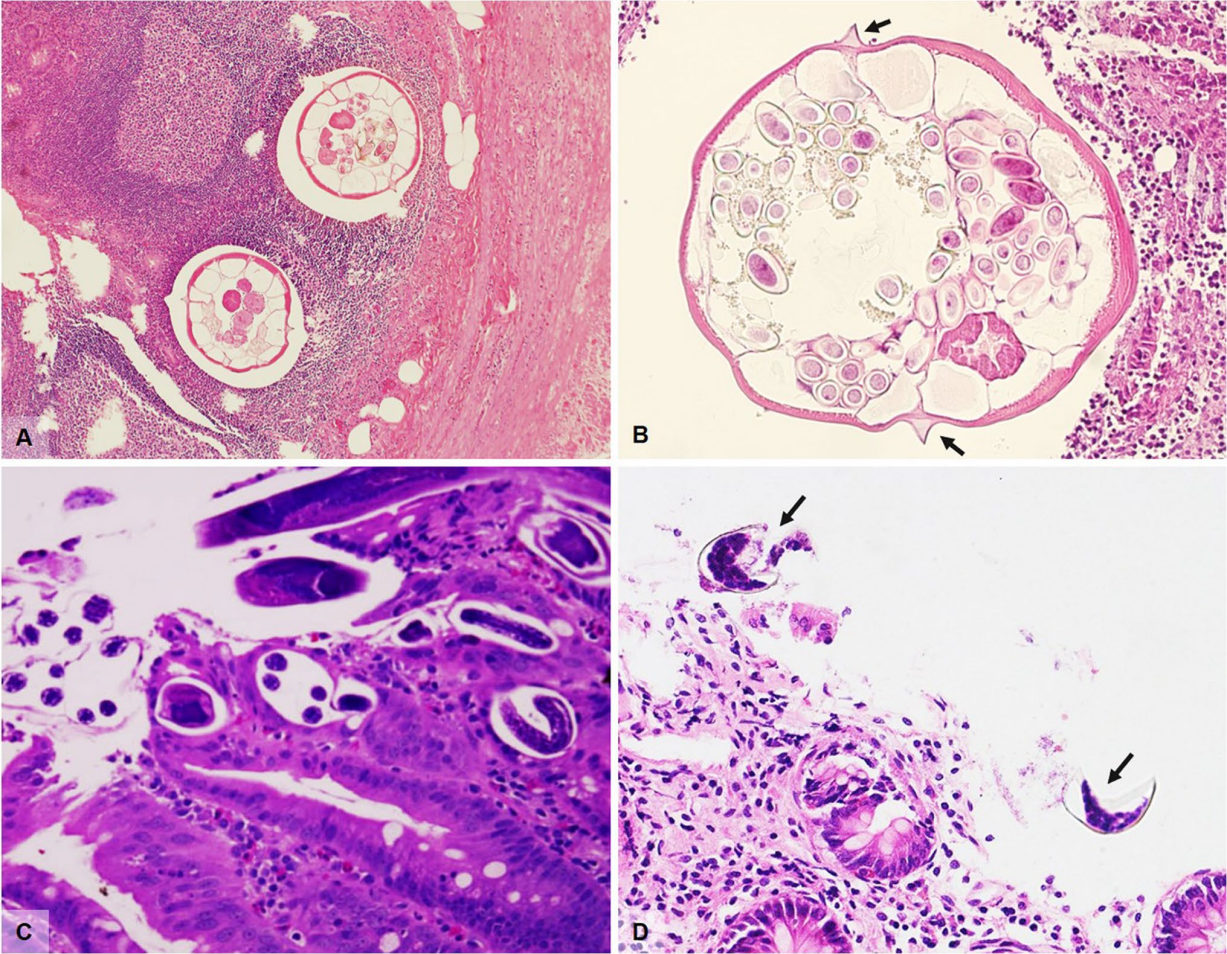
**A** *Enterobius vermicularis* in an appendiceal specimen (H&E, magnification 10 ×). **B** Cross section of *Enterobius vermicularis* showing typical lateral alae (arrows) (H&E, magnification 20 ×). **C** *Strongyloides stercoralis* in a duodenal biopsy characterized by larvae with sharply pointed, curved tails (H&E, magnification 20 ×). **D** *Diphillobothrium* eggs (arrows) in an otherwise normal colonic biopsy (H&E, magnification 20 ×)

In cross section, adult pinworms are easily recognizable even at low power, by their narrow lateral cuticular alae and visible internal organs; gravid females contain ellipsoid eggs characterized by a bilayered refractile shell. The morphologic features of the worm are characteristic, and the typical prominent lateral alae (Fig. 1A, B) permit distinction of *E. vermicularis* from *Trichuris trichiura* (whipworm), while isolated eggs must be differentiated from particles of vegetable matter. Both worms and eggs can calcify completely, making diagnosis more complex.

In case of invasive enterobiasis, an inflammatory reaction of variable intensity can be elicited, mainly composed of neutrophils and eosinophils, sometimes with mucosal ulceration and formation of granulomatous reaction with multinucleated giant cells.

### Strongyloides

Estimated *Strongyloides stercoralis* infections are as high as 100 million people worldwide [6]. *S. stercoralis* is a nematode predominantly found in tropical and some subtropical climates; however, widespread migration and immunosuppression are increasing the number of detected cases in developed countries also [7]. *S. stercoralis* follows a complex life cycle, infecting through the skin or oral mucosa and potentially leading to chronic disease that may last decades. After penetration, filiform larvae enter the venous system, reach the pulmonary alveoli, they migrate up the respiratory tract and larynx, and descend down the esophagus reaching the small intestine where they mature into adult worms. The adult is a parthenogenetic female able to deposit up to 50 eggs daily in the duodenal/jejunal lamina propria. Eggs hatch within the gut wall, excreting rhabditiform larvae that pass into stool or can mature into infective filariform larvae and disseminate to other organs [8]. Gastrointestinal symptoms begin about 2 weeks after infection from *S. stercoralis*, with larvae detectable in the stool after 3–4 weeks.

The term disseminated strongyloidiasis refers to the migration of larvae to organs beyond the range of the pulmonary autoinfective cycle while hyperinfection describes the syndrome of accelerated autoinfection, a life-threatening condition almost always occurring in immunocompromised individuals, such as organ transplant recipients, patients on long-term corticosteroid therapy, with hematologic malignancies, or with human T-lymphotropic virus type 1 (HTLV-1) infection, but curiously not associated with HIV [9]. Manifestations described in hyperinfection syndrome range from mild to severe, corresponding microscopically with mild initial mucosal congestion which may progress to edematous wall thickening, villous atrophy, or blunting and chronic inflammation. In more serious cases, dense chronic inflammation is accompanied by intense eosinophilic and neutrophilic infiltrate of the lamina propria and cryptitis. Granulomatous reaction surrounding organisms in degeneration may be seen. Severe infections, possibly associated with hyperinfection and sepsis, present with end-stage ulcerative enteritis. Parasites can be identified in the mucosa and rarely throughout the entire wall of the stomach, small bowel, and colon (Fig. 1C), as well as in lymphatic vessels and lymph nodes [10]. Rhabditiform larvae show typical, sharply pointed, sometimes curved tails, and measure approximately 180–380 μm in length, while eggs are oval (30–40 μm in diameter) and can be observed within the gravid females or in the glands/lamina propria of the gastrointestinal mucosa, in initial stages of disease. Adult female worms measure about 2.5 mm long and 0.05 mm wide. The anterior portion is characterized by a long esophagus while the posterior portion is occupied by intestines and reproductive organs.

### Diphyllobothrium

*Diphyllobothrium*, also known as fish tapeworm, is acquired through eating raw fish and is most common where freshwater fish consumption is highest, including Europe, and in particular Scandinavia and Northern Italy [11], as well as North America. Human diphyllobothriosis may be caused by as many as 14 different species with *D. latum* and *D. nihonkaiense* being the most frequent.

While the majority of infections are asymptomatic, approximately 20% of infections result in vague abdominal symptoms, such as diarrhea, abdominal pain or discomfort, such as well as fatigue, headache and allergic reactions. Fish tapeworm is a rare cause of B12 deficiency typically producing megaloblastic anemia in approximately 2% of infections, due to competition for vitamin B12 by the worm itself following parasite-mediated dissociation of the vitamin B12-intrinsic factor complex within the gut lumen [12].

The adult worm lives in the small bowel and can reach a length of over 20 m. There are no reported associated histologic changes in the gastrointestinal tract however tapeworm eggs may be rarely identified (Fig. 1D).

### Schistosoma

Schistosomiasis is caused by parasitic trematodes (flukes), endemic in sub-Saharan Africa, Middle East, South America, and the Caribbean [13]. Freshwater snails are intermediate hosts; after skin penetration and several maturation stages from egg to adult form,* Schistosoma* reach the liver and, by entering the blood stream, can migrate to the intestine, bladder, lungs, spleen, brain or spinal cord. S*chistosoma mansoni* and S*chistosoma japonicum* are responsible for the majority of intestinal infections [14].

Only a minority of patients with schistosomiasis are symptomatic. Pathologic lesions in gastrointestinal infections can involve any level [15], in particular the large bowel and rectum. At gross examination in acute cases, colonic mucosa appears edematous and congested with hemorrhages, erosions, superficial ulcers with a segmental or patchy distribution, requiring distinction from ulcerative colitis, Crohn’s disease, and ischemic colitis [16]. Inflammatory pseudopolyps, from few to several, sessile, pedunculated or cauliflower-like, ranging in size from 1 to ≥ 20 mm, may be observed in longstanding processes, and these are predominantly concentrated in the distal colon [17, 18].

**Fig. 2:**
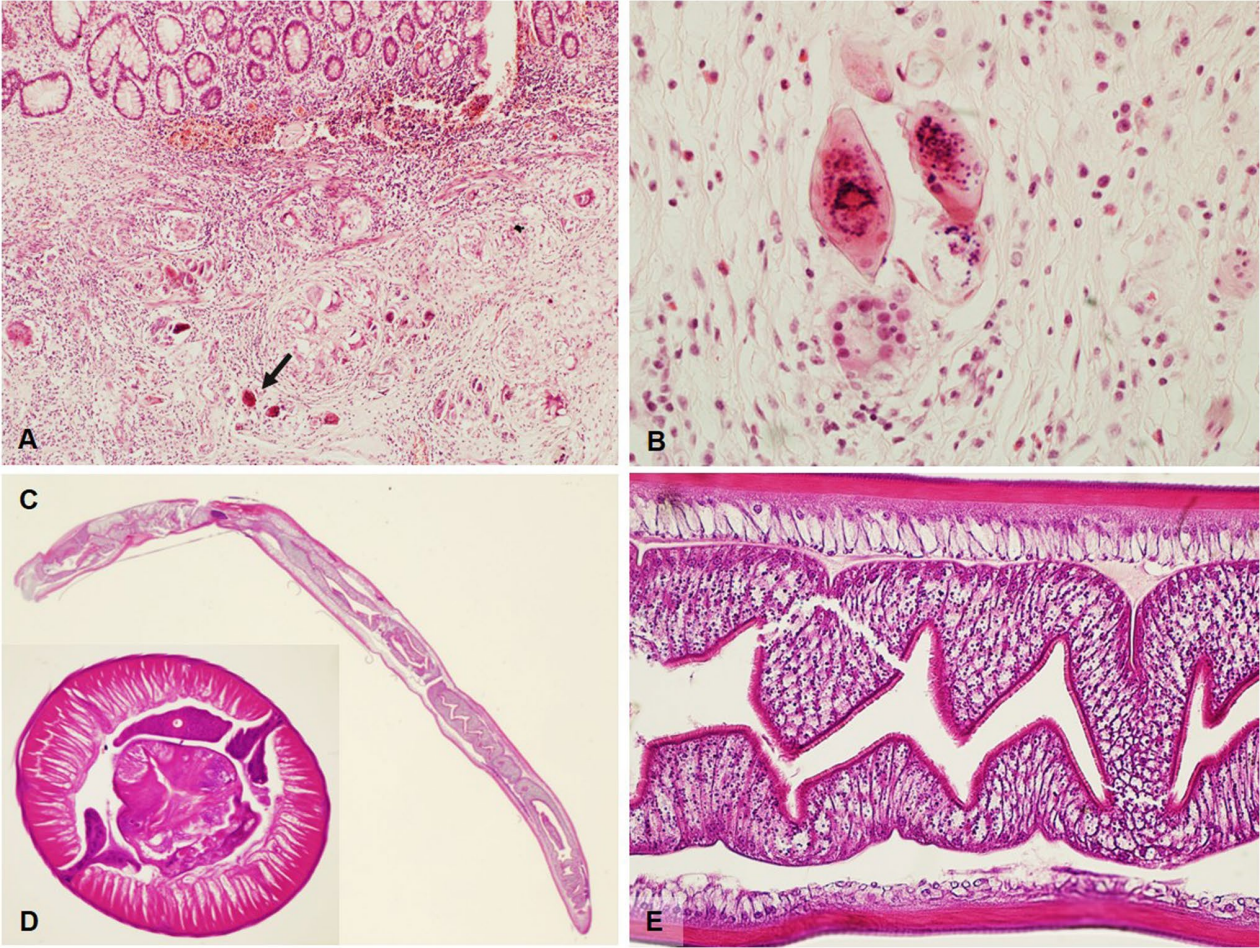
**A** Surgical resection specimen from a young male with ulcerative colitis who had just returned from holiday abroad showing multiple granulomas surrounding viable or calcified *Schistosoma Japonicum* eggs in the submucosa (H&E, magnification 10 ×). **B** *Schistosoma Japonicum* eggs with terminal spine (H&E, magnification 40 ×). **C** Longitudinal section of *Anisakis* larva found during colonoscopy in a patient with abdominal discomfort and recent consumption of raw anchovies (H&E, magnification 2 ×). **D** Cross section of *Anisakis* found in a surgically resected patient presenting with acute abdomen due to perforation and massive infiltration of eosinophils (H&E, magnification 40 ×). **E** Longitudinal section of *Anisakis* larva showing outer wall and internal digestive system (H&E, magnification 40 ×)

The pathognomonic microscopic finding is the presence of *Schistosoma* eggs, usually surrounded by a typically intense submucosal inflammation with peri-ovum granulomatous reaction (Fig. 2A, B) with prominent number of eosinophils, necrosis, cellular debris and granulation tissue. Overlying mucosa may be either ulcerated and denuded or hyperplastic with formation of pseudopolyps and polyps showing ulceration, gland distortion, mucin depletion, and/or dysplasia. Splendore–Hoeppli phenomenon, which is the formation of radiate eosinophilic material surrounding microorganisms such as fungi and parasites, is sometimes observed. Fibrosis increases with disease progression, possibly leading to luminal narrowing and obstruction of the bowel, and inflammatory infiltration becomes mononuclear, with macrophages and multinucleated giant cells. Eggs can become completely calcified, with total disappearance of inflammation.

### Anisakis

Anisakidosis is habitually described as an infection occurring in Japan, most commonly sustained by *Anisakis simplex* and *Pseudoterranova decipiens*; in the last years, parallel to novel fads for raw or undercooked marine fish or squid consumption with sushi, sashimi and ceviche, cases of gastrointestinal infections due to this nematode have been described in other countries [19]. *Anisakis* worms (white to pinkish vermiform nematode, 10 to 29 mm long and 0.5 mm width) are recognizable at endoscopy and sometimes at gross examination, embedded in the mucosa, most frequently of the stomach, although small intestine, appendix, and rarely right colon can be involved as well. Gastric manifestations are the most readily encountered in Japanese patients, whereas intestinal disease is more common in Europe. Extra-gastrointestinal complications, resulting from larval transmural penetration of the stomach or intestine, and allergic syndromes are less frequent.

Macroscopically, gastric folds may appear thickened and inflamed [20] while the histopathologic lesions have been traditionally classified into different steps, depending on timing and intensity of infection [21]. Initially, infiltration by neutrophils with few eosinophils and foreign body giant cell reaction are seen. Larvae with prominent multilayered cuticles, distinctive Y-shaped lateral chords but lacking lateral alae or wings are seen in tissue sections, particularly resection specimens [22] (Fig. 2C–E). Within the first week of an acute intestinal infection, the eosinophilic infiltrate may become massive, associated with lymphocytes, monocytes, neutrophils, and plasma cells, as well as edematous thickening of the submucosa. Worm degeneration may induce neutrophilic abscess formation with surrounding histiocytes and fibrosis, and over time, a granulomatous reaction with central necrosis and eosinophilic infiltrate may prevail. In these latter cases, degenerated larvae are not easily observed but at least some portions may be detectable with perseverance and further sections.

## Protozoa

Protozoa are one-celled organisms that can affect humans, causing possible serious infections. Transmission of intestinal-based protozoa occurs through contaminated food/water or from interpersonal contact. Only the most frequent protozoa are discussed below.

### Entamoeba

One of the leading causes of parasitic death worldwide, accountable for more than 50,000 deaths a year, *Entamoeba histolytica* is a protozoan with marked tissue-invasive properties [23]. This human-only parasite is endemic in areas from Central and South America, Africa, and Southeast Asia, whereas in Western countries, it is more common among travellers or immigrants [24]. While *E. histolytica* was formerly believed to infect about 10% of the world population, it was later recognized that up to 90% of patients were colonized by other nonpathogenous species, such as *E. dispar*, which are morphologically indistinguishable [25, 26].

The vast majority of cases are asymptomatic; however, amoebic colitis often presents with subacute, gradual onset of watery or bloody diarrhea, abdominal pain, anorexia, and weight loss; infrequently, patients develop fulminant colitis with fever, profuse bloody diarrhea and signs of peritonitis, a condition usually associated with bowel necrosis, perforation and toxic megacolon, with a high mortality rate (> 40%). The most common extra-intestinal manifestation is amoebic liver abscess, which can present months to years after primary infection. Liver abscesses may rupture through the diaphragm, thus giving rise to pleuropulmonary or pericardial involvement, the latter being a rare event associated with high mortality (30%). Alternatively, amoebas may disseminate through hematogenous spread to distant structures, such as the central nervous system [23, 24, 26].

**Fig. 3:**
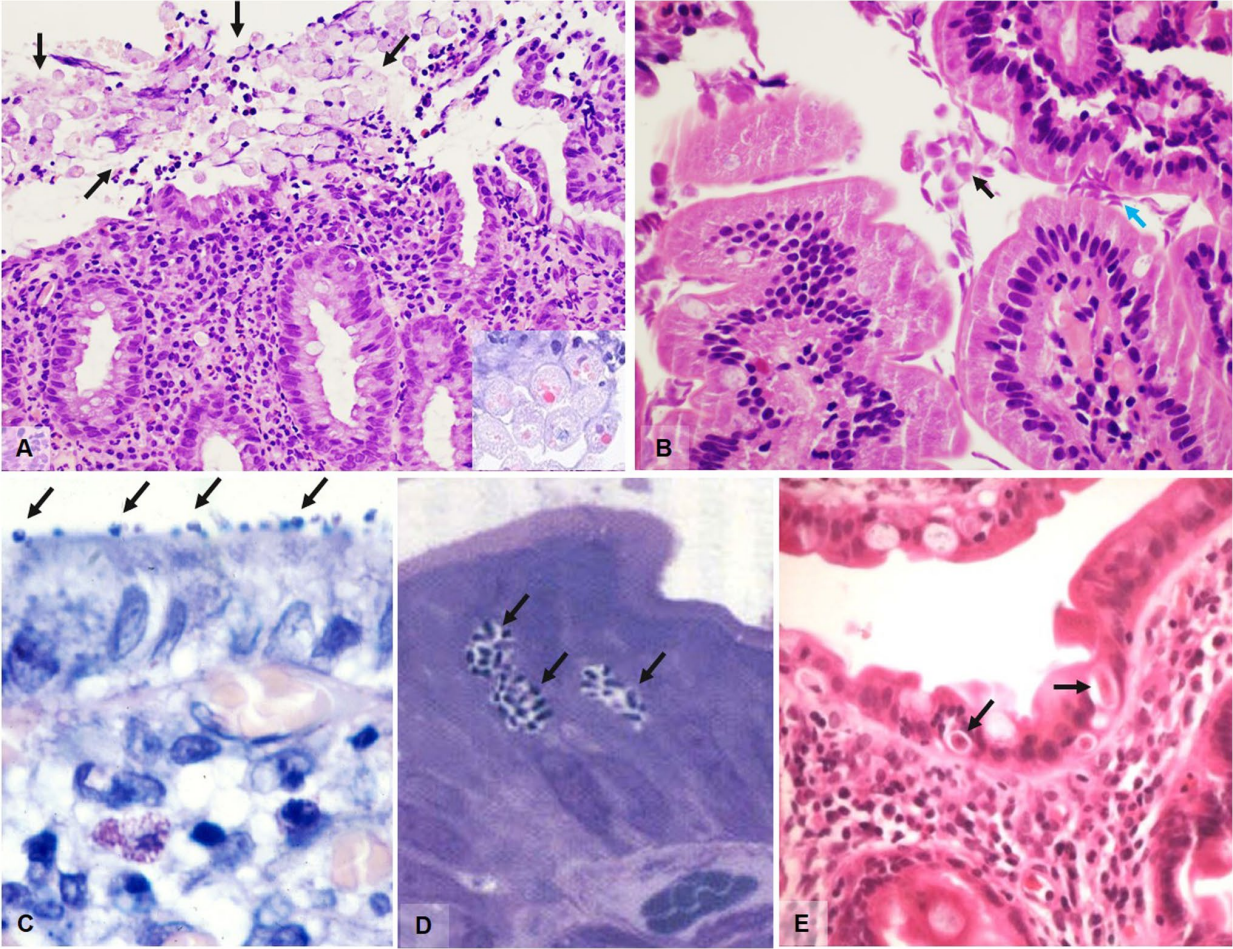
**A** Numerous *Entamoeba histolytica* (black arrows) on the surface of a colonic biopsy with erosion and inflammation (H&E, magnification 20 ×). Insert shows haemophagocytosis in *Entamoeba* (H&E, magnification 40 ×). **B** *Giardia lamblia* on the surface of a duodenal biopsy. The black arrow shows pear-shaped trophozoites while the cian arrow shows typical nail clipping appearance (H&E, magnification 60 ×). **C** *Cryptosporidium parvum* in an endoscopic biopsy from a young female with AIDS showing small basophilic spherules on the surface (arrow) (Giemsa stain, magnification 100 ×). **D** *Microsporidia* seen as round oval basophilic, supranuclear bodies in the cytoplasm of enterocytes (arrows) (Toluidine blue, magnification 200 ×). **E** *Isospora belli* found in a duodenal biopsy from an AIDS patient, as banana shaped or oval organisms found between enterocytes with peripheral halo (H&E, magnification 60 ×)

Intestinal amebiasis usually affects the proximal colon but also other intestinal tracts can be involved, from the distal ileum to the anal canal. Bowel invasion leads to the characteristic flask-shaped ulcer, due to narrow disruption through the mucosa and submucosal lateral expansion. Trophozoites tend to localize on the advancing edges of the ulcer but may be seen through the whole intestinal wall. Chronic active inflammatory response, edema and congestion, necrosis, and goblet cell depletion are usually seen in the mucosa. Untreated/inadequately treated patients may develop amebomas, exophytic masses resulting from continuous granulation tissue formation and fibroblastic proliferation. Biopsy diagnosis may be challenging and is based on the recognition of trophozoites: round to ovoid-shaped, ranging from 6 to 40 µm and having a PAS-positive cytoplasm with granular appearance (Fig. 3A and insert). Erythrophagocytosis, distinctive of *E. histolytica*, can be demonstrated with iron-hematoxylin staining [27]. Nuclear morphology is one of the main characteristics which helps in distinguishing a macrophage that has ingested erythrocytes from an amoeba. Macrophages show multiple clumps of chromatin while amoebas have a karyosome that is usually round and the chromatin is dispersed with minimal to no clumping.

### Giardia

*Giardia lamblia* is a flagellate protozoan responsible for giardiasis, the most common protozoal intestinal infection in humans worldwide, with an estimated 280 million cases per year [28, 29]. Despite *Giardia* being a ubiquitous parasite, its prevalence in humans ranges from about 2–7% of the population in developed countries (giardiasis is more common in the USA compared to Europe), to 30%, and possibly higher, in developing countries from South America, Africa and Asia [28, 30]. Immunodeficient patients, both children and adults, are especially at risk of contracting giardiasis and are more likely to exhibit symptoms.

Giardiasis is often asymptomatic, with 50 to 75% of patients showing no symptoms and self-eradication of infection [28]. Acute giardiasis usually presents after 1–3 weeks of incubation with watery diarrhea, abdominal pain and cramping, flatulence, anorexia, weight loss, fatigue, and malaise while fever, vomiting, and bloating are less common [31, 32]. Chronic giardiasis is characterized by intermittent, foul-smelling diarrhea, malabsorption, and failure to thrive in children [29].

*G. lamblia* is mainly found in the duodenum, but jejunal, ileal, gastric, and colonic localization have been reported, if seldomly. Giardiasis is typically a noninvasive infection, with trophozoites attaching to enterocyte microvilli and proliferating without penetrating into the intestinal wall [29]. Possible histopathologic findings include lymphocytosis, inflammation, villous atrophy and crypt hyperplasia but, more often than not, the mucosa is morphologically normal [29]. *Giardia* trophozoites are pear-shaped and binucleated but appear elliptical when seen in profile (typical “nail clipping” appearance), and measure 10–20 µm in length (Fig. 3B).

### Intestinal *Coccidia* and related organisms

*Coccidia* are a large group of protozoan parasites infecting the intestinal mucosa of both humans and animals. Their life cycle comprises two phases and can be found as parasitic sporozoites in the intestine or as free-living oocysts (encapsulated zygotes), usually discharged in the feces of contaminated animals/humans. These parasites are especially dangerous to immunocompromised patients, where they can lead to severe diarrhea and death. With regard to C*ryptosporidia*, the most frequent species to infect man are *C. parvum* and *C. hominis*, and these are intracellular obligate parasites which can be transmitted through the fecal–oral indirect route or interhuman contact [33–35]. Colonization is predominantly in the proximal part of the small bowel and gastric involvement is possible in areas of intestinal metaplasia. *Cryptosporidia* can be found on the luminal surface of enterocytes as small basophilic spherules measuring approximately 4–5 µm as non-specific mucosal inflammation may be found (Fig. 3C).

*Microsporidia*, once classified as protozoa, are a large group of unicellular intracellular parasites closely related to fungi; the most frequently pathogenic species are *Enterocytozoon bieneusi*, and some *Encephalitozoon* species [33]. They are difficult to spot as their location is supranuclear, intracytoplasmatic, and appear as round/oval, basophilic bodies with clear halos measuring between 1 and 2 microns depending on the species (Fig. 3D). The intestinal mucosa may show minimal to no damage or a range of villus atrophy, epithelial disarray, and increased intraepithelial lymphocytes. Giemsa or Warthin-Starry stains may prove helpful even though diagnosis is nowadays based on molecular diagnostic tests.

While *Cytoisospora Belli* (Fig. 3E) is one of the most common coccidian parasites infecting HIV/AIDS patients in developing countries (Africa, Asia, and Latin America), it is rarely found in the Western world. *Cytoisospora* infects enterocytes of the small bowel and can be seen as vacuoles with ovoid or banana shaped organisms, similar in size to enterocyte nuclei. Recently, *Cytoisospora* infection has been described in gallbladders from immunocompetent patients with acalculous cholecystitis [36]; however, electron microscopic and molecular analyses have shown that in many cases, these are actually inclusions of condensed cytoplasmic material mimicking organisms [37].

### Diagnosis of infectious causes in practice

Diagnosis of helminths or protozoa in the gastrointestinal tract may be a surprise for the general pathologist and may require the consultation with a clinical microbiologist or infectious disease pathologist to define with precision and certainty the exact type of parasite. Parasites recognition only rarely relies solely on histology; in clinical practice diagnosis is usually performed by stool exam for ova or parasites. Furthermore, molecular methods using multiplex molecular platforms are now available for protozoa identification [38].

## Drugs

While numerous treatments can cause gastrointestinal injury [39, 40], in the majority of cases, only the drug’s effect is histologically evident while the drug itself is not commonly identified. Rarely, however, substances constituting the drug, including fillers, can be histologically detected in the form of aggregates of amorphous material, crystals, or resins, some of which may be associated with tissue damage.

### Iron pill

**Fig. 4:**
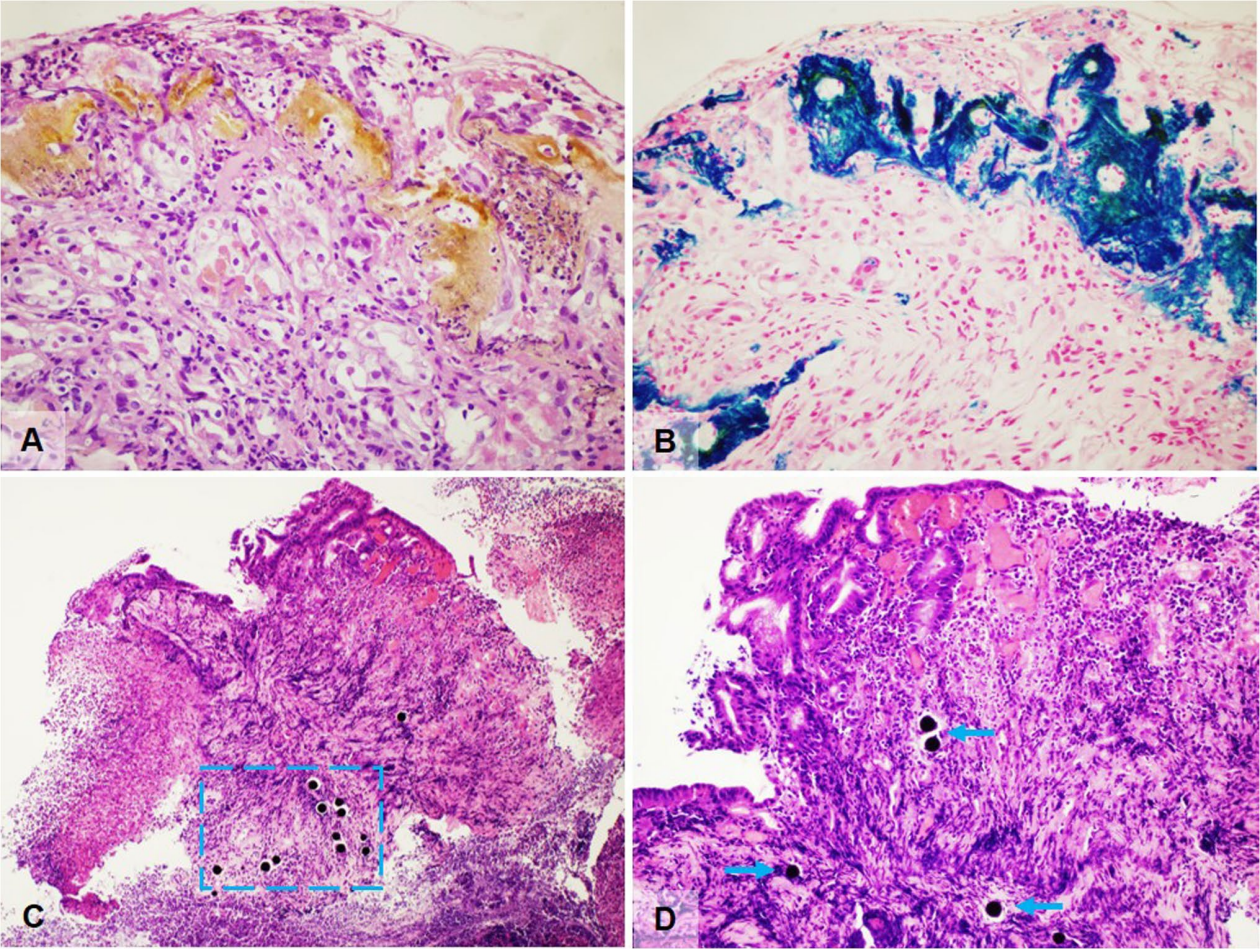
**A** Iron pill gastritis with gastric erosion with presence of interstitial aggregates of amorphous yellow-ochre substance together with neutrophils in a patient taking iron supplements for anemia (H&E, magnification 40 ×). **B** Iron pill gastritis stained with Perls Prussian blue stain demonstrating the ferrous nature of the deposits (Perls Prussian blue, magnification 40 ×). **C** Yttrium-90 microspheres in gastric biopsies with ulcers, active inflammation and epithelial damage in a patient treated by radioembolization with yttrium-90 microspheres for liver cancer. Lesions are coherent with radiation injury and black, spherical foreign bodies are seen in the mucosa and submucosa, within a cian dashed line box (H&E, magnification 10 ×). **D** Higher magnification of yttrium-90 microspheres (arrows) (H&E, magnification 20 ×)

Iron pill supplements, commonly used in iron deficiency anemia, may cause mucosal damage and may be deposited, becoming histologically visible, in the upper gastrointestinal tract. Most frequent sites include the stomach (as well as esophagus and duodenum), leading to iron pill-induced gastroduodenopathy [41, 42] with deposits of brown-black crystalline hemosiderin (Perl’s positive) on the epithelial surface, and these may be associated with erosion, fibrosis, inflammation, and foreign body reaction (Fig. 4A, B). Iron-pill-induced mucosal damage is characterized by predominant extracellular deposition of iron on the epithelial surface with only focal intracellular deposits in the epithelium, blood vessels, and macrophages [43, 44]. Iron deposits should be distinguished from non-specific intracellular iron deposition within macrophages, stromal cells, and epithelium secondary to prior mucosal hemorrhage and diffuse iron deposition (in mucosal glands), due to systemic iron overload (e.g., in hereditary hemochromatosis and multiple blood transfusion) [45].

### Yttrium beads and SIRT spheres

Treatment modalities for liver neoplasia include yttrium-90 radiotherapy, which is a method of targeted high-dose intra-arterial radiation delivered by using glass beads (theraspheres) and resin (SIRspheres) microspheres. Radioactive spheres can embolize/migrate to extrahepatic locations, including the upper gastrointestinal tract [46]. Patients may develop gastrointestinal injury after treatment (between 3 and 29% of treated patients), with symptoms such as nausea/vomiting, odynophagia, hematemesis, and melena, presenting between 10 days and 5 months postprocedure [47]. Lesions can occur mostly in the gastric antrum, pylorus, and duodenum and biopsies show ulcers, erosions with active inflammation and possible ischemic changes, and epithelial damage (nuclear and cytoplasmic enlargement, hyperchromasia, bizarre nuclei) coherent with radiation injury. The characteristic 20–30-µm spherical foreign bodies (black/deep purple SIRspheres; colorless and refractile theraspheres) are seen within the granulation tissue/ulcer [48] (Fig. 4C, D). A chronic course of the ulcers, dating for years, with possible stenosis, is also possible [49].

### Resins

Resins are nonabsorbable drugs, used for ion exchange in the gastrointestinal tract in various chronic conditions. Three types of resins are identifiable: sodium polystyrene sulfonate (kayexalate), sevelamer, and bile acid sequestrants (including cholestyramine, colesevelam, and colestipol) [50].

**Fig. 5:**
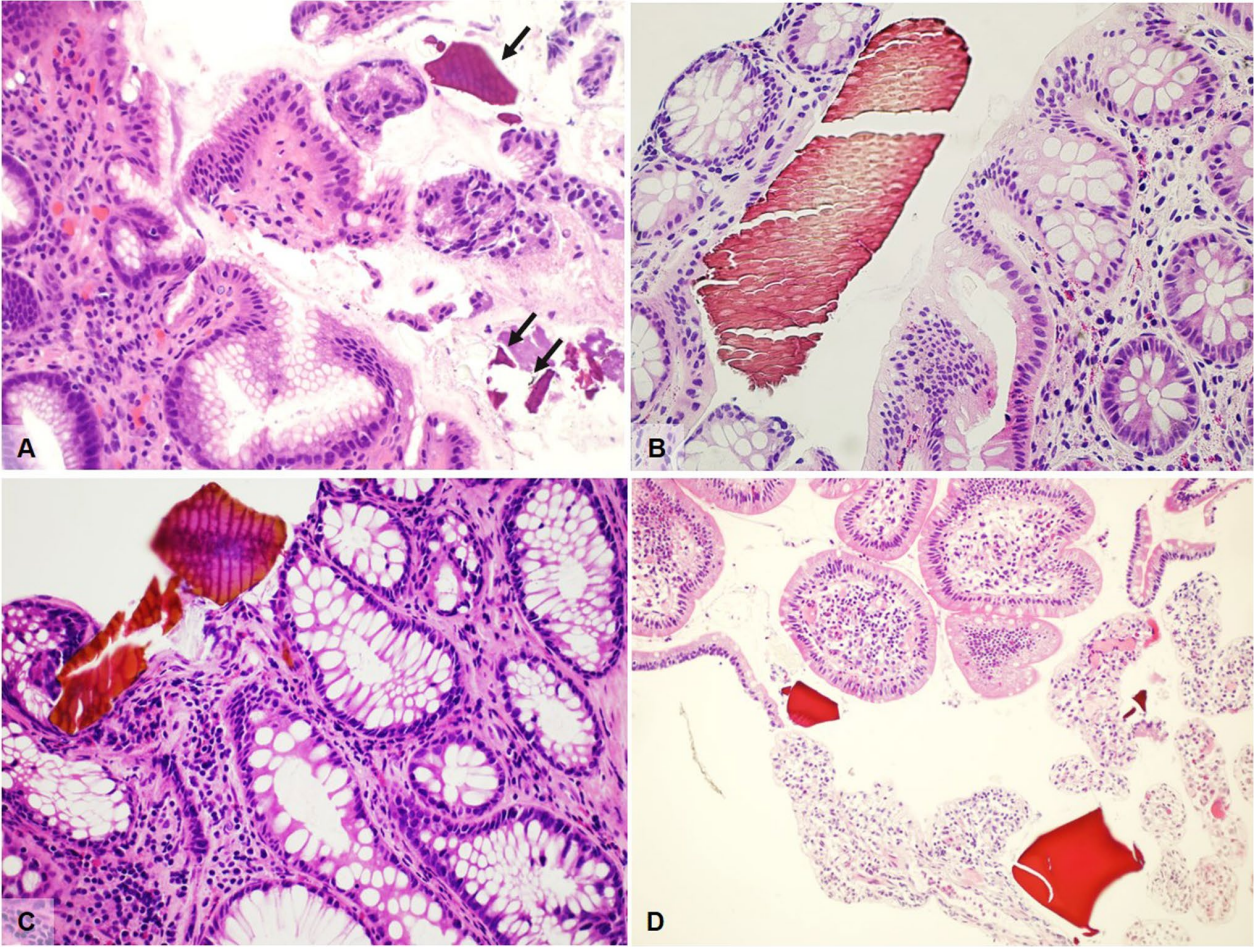
**A** Kayexalate resins in a gastric biopsy from a young patient on renal transplant list. The stomach shows violet–purple color deposits with a typical fish-scale appearance (arrows) overlying an eroded mucosa with marked reactive changes (H&E, magnification 40 ×). **B**, **C** Sevelamer deposits in biopsy samples from two different patients showing the typical two-toned pink and yellow colour with fish scale pattern (H&E, magnification 40 ×). **D** Bile acid sequestrants (BASs) deposits with characteristic polygonal shape, with a smooth and glassy texture, eosinophilic staining, and no fish-scale pattern (H&E, magnification 20 ×)

*Sodium polystyrene sulfonate* (kayexalate) is a cation-exchange resin routinely used in the management of hyperkalemia in patients with chronic kidney disease. Its use, both in preparations containing sorbitol (which is often the main cause of injury) or without sorbitol, is associated with gastrointestinal adverse events, although direct pathogenicity is rare. Adverse events, some of which are potentially fatal, include ulceration, colonic transmural necrosis, or perforation [51, 52]. Upper gastrointestinal damage is less frequently reported, can involve the esophagus, stomach and duodenum, and, in some cases, radiologically and endoscopically mimicks esophageal carcinoma or bezoar. Mucosal ulcers and erosion are histologically evident in almost all cases, but transmural necrosis, perforation, and death are fortunately extremely rare [53]. Histologically, kayexalate is recognizable on the surface or embedded in erosions or ulcers; it appears as a violet–purple colored substance with a typical fish-scale appearance (Fig. 5A).

*Sevelamer* is an anion-exchange resin used in patients with chronic renal disease to treat hyperphosphatemia. Mucosal damage, especially in the lower gastrointestinal tract is reported, spanning from nonspecific colitis with crypt distortion to ulcers and colonic perforation, even though a direct role of sevelamer in colonic injury is as yet undemonstrated. Histologically, sevelamer shows a two-toned color with a rusty yellow background and bright pink linear accentuation on H&E. Furthermore, sevelamer shares with kayexalate, a similar fish scale pattern [54] (Fig. 5B, C).

Bile acid sequestrants (BASs) include different resins (cholestyramine, colesevelam, colestipol, and others) used to treat hypercholesterolemia, dyslipidemia, or bile acid-mediated diarrhea [55]. BAS resins are frequently identified in gastrointestinal specimens, sometimes associated with chronic or acute inflammation, erosion, or ulcers. BASs are indistinguishable from one another; they appear polygonal in shape, with a smooth and glassy texture, variable eosinophilic staining, and lack the regular fish-scale pattern seen in other resins (Fig. 5D).

### Pharmaceutical fillers

Fillers are insoluble, nonabsorbable powders added to pharmaceuticals to help with the manufacturing/stabilization of the active pharmaceutical ingredient, without modifying its effectiveness. Fillers are widely used in many over-the counter and prescription medications, as well as vitamins and processed food products. The most frequently used are crospovidone and microcrystalline cellulose (MCC), and these have been identified in 0.3–9.0% of gastrointestinal surgical specimens [56, 57].

**Fig. 6:**
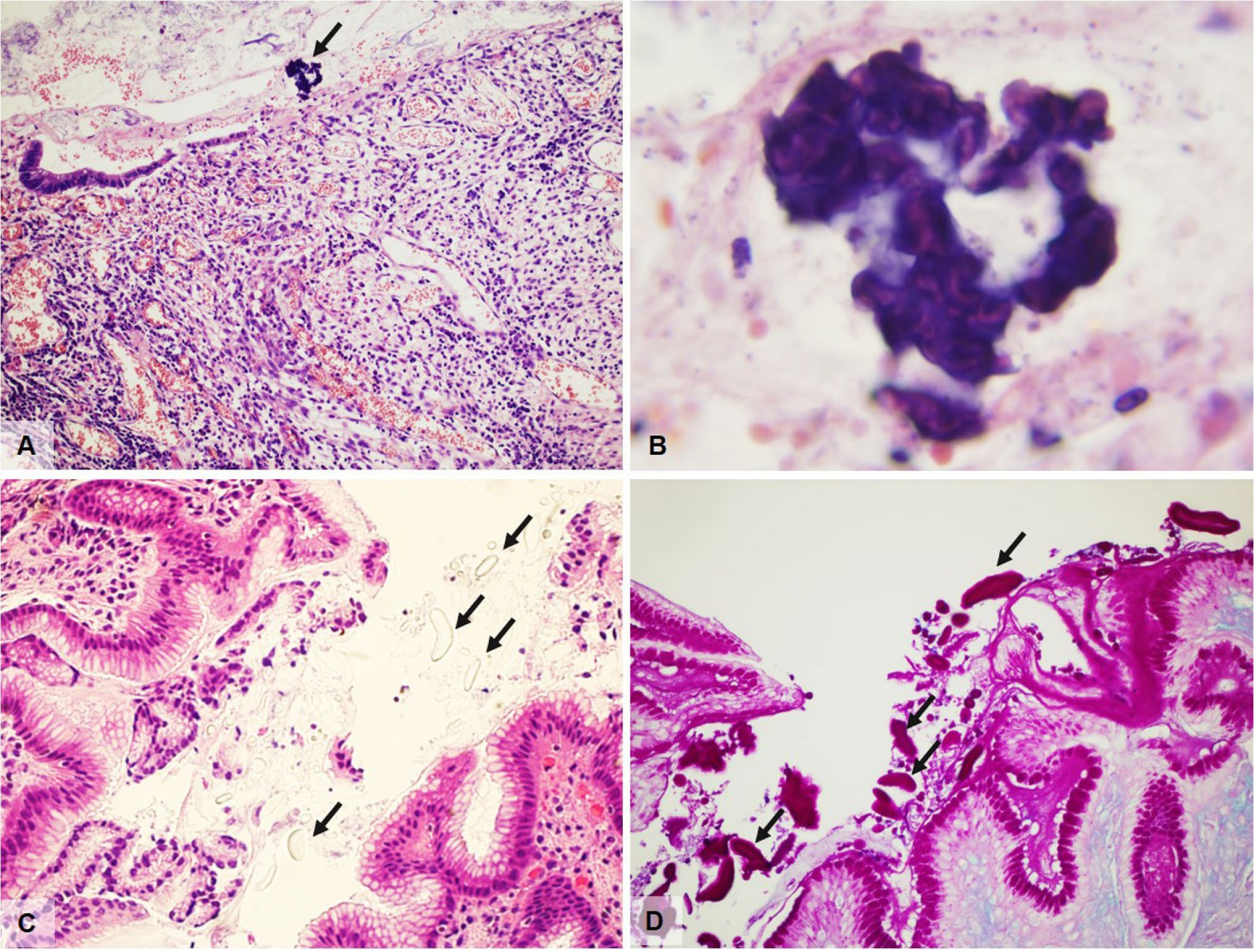
**A** Left colectomy showing ulcerations due to ischemic colitis and residue from the pharmaceutical filler crospovidone (arrows) (H&E, magnification 20 ×). **B** Higher magnification of the previous image showing deeply basophilic coral like particle of crospovidone (H&E, magnification 100 ×). **C**, **D** Endoscopic biopsies from the stomach showing microcrystalline cellulose filler on the surface epithelium which is transparent on H&E (**C**) and magenta on PAS stain (**D**) (magnification 40 ×)

Crospovidone and MCC are generally not associated with mucosal damage in the gastrointestinal tract; they can be found free-floating on the surface epithelium, or they can become an indirect sign of perforation when they are found on the peritoneal surface. Crospovidone (Polyvinylpolypyrrolidone) appears as deeply basophilic coral-like or sponge shape particle on the surface of colonic mucosa (Fig. 6A, B) while MCC is rod-shaped, transparent on H&E, light purple with PAS stains and birefringent under polarized light (Fig. 6C, D).

## Seeds

Partially digested plant remnants are frequently found in both biopsy and surgical gastrointestinal specimens. They are easily recognizable by the polygonal shape of cells, by the thick cell wall and the presence of starch granules. Sometimes they can be surrounded by a granulomatous reaction with giant cells, especially in case of complicated diverticulitis or acute appendicitis, but also in case of ulcerated and perforated neoplasms.

**Fig. 7:**
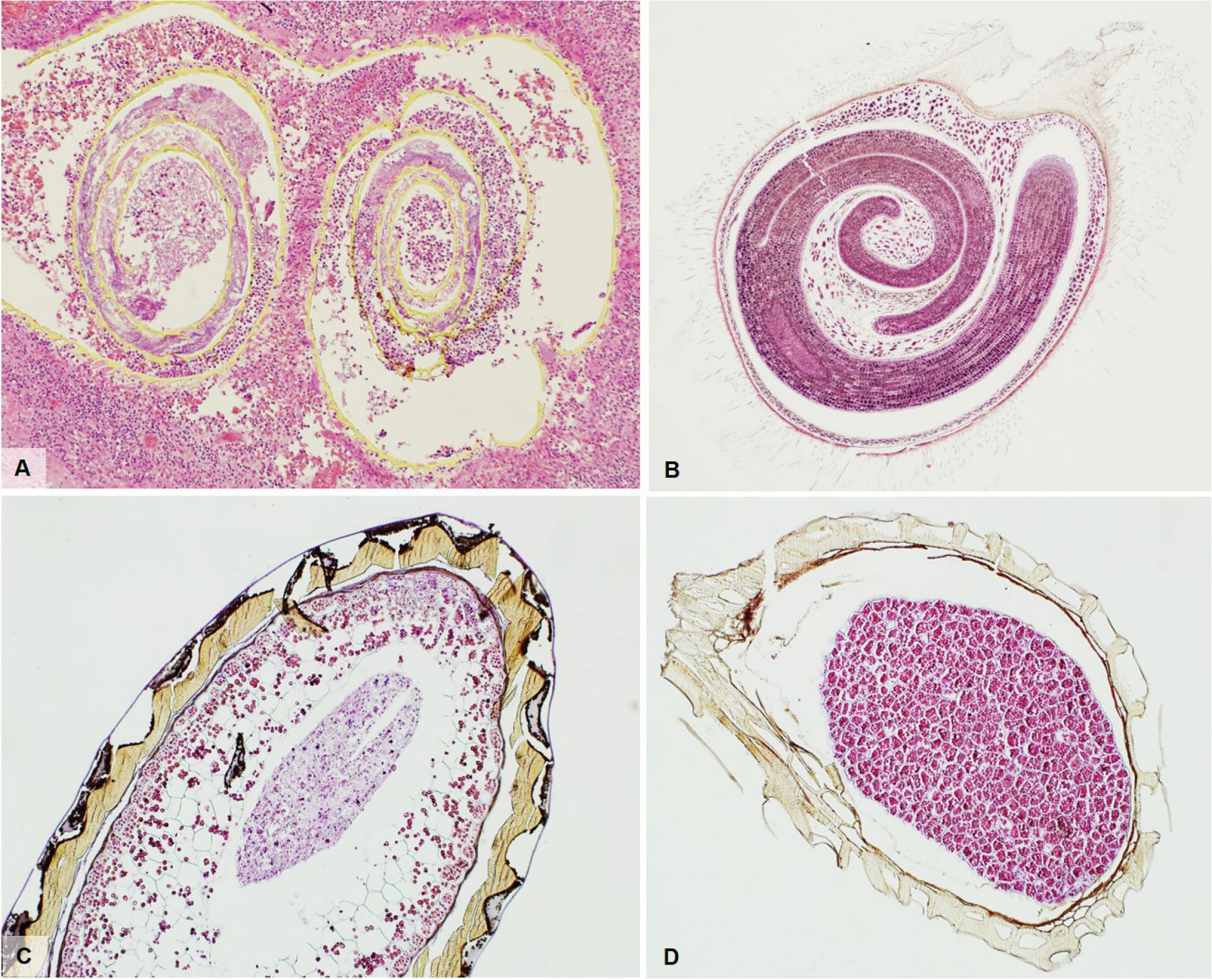
**A** Left colectomy for acute diverticulitis showing, within an inflamed diverticulum, yellow-orange coloured tomato-peal with festooned appearance (H&E, magnification 10 ×). **B** Tomato seed with outer seed coat with numerous filaments and inner embryo (H&E, magnification 4 ×). **C** Kiwi seed (H&E, magnification 10 ×). **D** Blueberry seed (H&E, magnification 20 ×)

Some plant residues, on the other hand, have a particular configuration and their recognition and correct attribution can be more intriguing. Among these, tomato peel, which is frequently found in patients who follow a Mediterranean diet, is recognized for its yellow-orange color, due to the richness in carotenoids, and for its festooned appearance [58] (Fig. 7A).

Many different types of seeds can be found in all gastrointestinal samples, as they often remain undigested due to the particular resistance of the seed’s external cuticle to digestion. Various types of seeds have been reported in biopsy samples, polypectomy specimens and in various types of surgical specimens including appendicectomies, diverticular disease associated resections and cancers [59, 60]. Among the most frequently found seeds, tomato seeds deserve more careful attention due to their complex morphology with glycoprotein-rich villi on the surface (which facilitate adhesion to the intestinal mucosa) making a misdiagnosis as parasites possible (Fig. 7B). Other frequently found seeds are kiwi (Fig. 7C) and berries (especially blueberries and raspberries) (Fig. 7D), but almost any type of ingested small seed can be found. Their specific identification, though useless from a histopathological diagnostic point of view, is not always easy due to the lack of iconographic texts available in the medical scientific literature.

## Pollutants

In the context of histological or cytological samples, various pollutants can be found, randomly ending up in the sample. Pollen, mites and, rarely, even midges fall into this category.

**Fig. 8:**
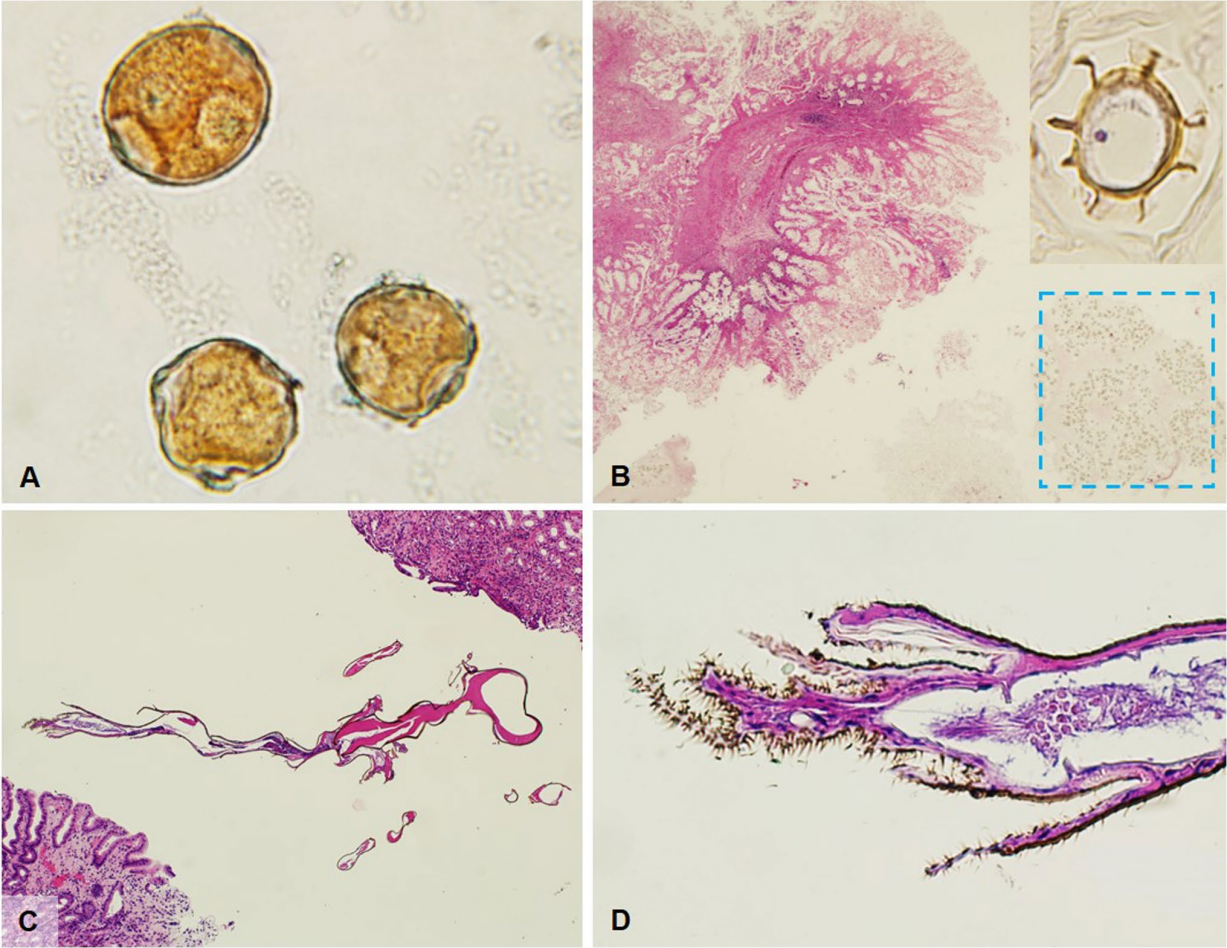
**A** Pollen found in a cytology specimen which had been dried near an open window in spring (H&E, magnification 10 ×). **B** Autolytic colorectal adenoma recovered by the patient from the feces and placed in a nonsterile container. Within the dashed-line box numerous *acari* can be found; the insert shows typical oval shape with external carapace and legs ((H&E, magnification 2 × and 100 ×). **C** Accidently paraffin embedded midge in gastric biopsies (H&E, magnification 10 ×). **D** Higher magnification of the caudal part of the midge showing hairy portions of the body (H&E, magnification 40 ×)

Pollen can frequently be found in cytological preparations left to dry in the air. Pollen has dimensions ranging from 10 to 100 microns (Fig. 8A). Typical features are the shape, generally rounded or oval, the presence of a very resistant outer layer, the exine, and the exine ornamentation showing an enormous variety of patterns (e.g., psilate, microechinate, foveolate, etc.).

*Acari*, part of the superorder Acariformes including diverse groups of mites (Astigmatina), are small, eight-legged arthropods (arachnids) which can be frequently found in stored products, fur, feather, and dust. A peculiar case which caught our attention was the incidental finding of numerous acari in a polypectomy specimen. The patient, who had undergone endoscopic polypectomy but whose polyp had not been retrieved, noticed the polyp in his faeces the following day; the polyp was recovered, placed in a non-sterile container and taken to the pathology lab. Acari are oval-round in shape, with an external carapace and four pair of legs (Fig. 8B).

*Midges* can contaminate histologic samples during different stages of processing, and represent an unexpected and intriguing finding. Their complex morphology and the recognition of specific structures such as the buccal apparatus or the hairy paws, easily allow recognition (Fig. 8C, D).

## Conclusions

Weird and wonderful findings in the gut lumen sometimes pose difficulties for the pathologist particularly as a clear and useful iconography is often missing. We hope that the above descriptions and figures will aid the young, and not so young, pathologist in identifying “stranger things” in the gut.

## Data Availability

Not applicable.

## Declarations

The study was conducted in accordance with the ethical standards of the Helsinki Declaration (1964, amended in 2008) and of the World Medical Association.
